# Supplementary material for: An approach to MOFaxanes by threading ultralong polymers through metal–organic framework microcrystals
Source: Nat Commun. 2023 Jun 9;14:3241. doi: 10.1038/s41467-023-38835-5 (PMC10256729; doi:10.1038/s41467-023-38835-5)
Supplement: Supplementary file 1 — Supplementary Information [file 41467_2023_38835_MOESM1_ESM.pdf]

**Supplementary Information**

# An approach to MOFaxanes by threading ultralong polymers through metal–organic framework microcrystals

Tomoya Iizuka, Hiroyuki Sano, Benjamin Le Ouay, Nobuhiko Hosono,* Takashi Uemura*

Correspondence to: nhosono@g.ecc.u-tokyo.ac.jp, uemurat@g.ecc.u-tokyo.ac.jp

**Table of contents**


**I. Synthesis and characterization of $[Cu_2(bdc)_2(bpy)]_n$ (1)**

1. PXRD patterns of **1** and **1'** (Supplementary Fig. 1, 2) ........ 3
2. Investigation of the MOF crystal size and size comparison between MOF and PEO (Supplementary Fig. 3, 4) ........ 5
3. Thermogravimetric analysis of guest included **1** (Supplementary Fig. 5) ........ 7
4. Discussion about the driving force of PEO penetration in **1** (Supplementary Fig. 6–8) ........ 8

**II. Discussion about the infiltration process of PEO into 1**

1. In-situ PXRD analysis using end-capped **PEO20k** with bulky *tert*-butyldiphenylsilyl (TBDPS) group (Supplementary Fig. 9) ........ 11

**III. Structural investigation of polypseudoMOFaxane**

1. PXRD patterns of **1/PEO4M** composites with less PEO loading amount (Supplementary Fig. 10) ........ 12
2. Estimation of the coverage of polypseudoMOFaxane (Supplementary Fig. 11) ........ 13
3. Particle size distribution analysis of polypseudoMOFaxane (Supplementary Fig. 12) ........ 14
4. AFM imaging of polypseudoMOFaxane (Supplementary Fig. 13, 14) ........ 15

**IV. Effect of polyMOFaxane structure on the properties of 1/PEO composites**

1. Investigation of the effect of the polypseudoMOFaxane structure on the crystallization rate of PEO (Supplementary Fig. 15–19, Supplementary Table 1) ........ 17
2. Solvent washing experiment on **1/PEO4M** composite to investigate kinetic prevention of the unthreading reaction (Supplementary Fig. 20–23) ........ 23
3. Uniaxial tensile stress-strain analysis on the **1**/PEO composites (Supplementary Fig. 24, 25, Supplementary Table 2 and 3) ........ 27

**V. Supplementary References** ........ 30

## I. Synthesis and characterization of $[Cu_2(bdc)_2(bpy)]_n$ (1)

### 1. PXRD patterns of 1 and small-sized 1 (1')

The small-sized **1** crystal (**1'**) was synthesized by the same protocol for **1**, except that acetic acid was not added. The resultant product was washed with *N*,*N*-dimethylformamide (DMF) and methanol, then evacuated at 130 °C for 16 h to afford **1'** with diameter of ~210 nm.

PXRD patterns of activated **1** and **1** after immersion into methanol (**1**⊃methanol) are shown in Supplementary Fig. 1. Simulated patterns of **1**-*op* and **1**-*cp* are also given. The activated **1** has closed phase while **1**⊃methanol has open phase structure.

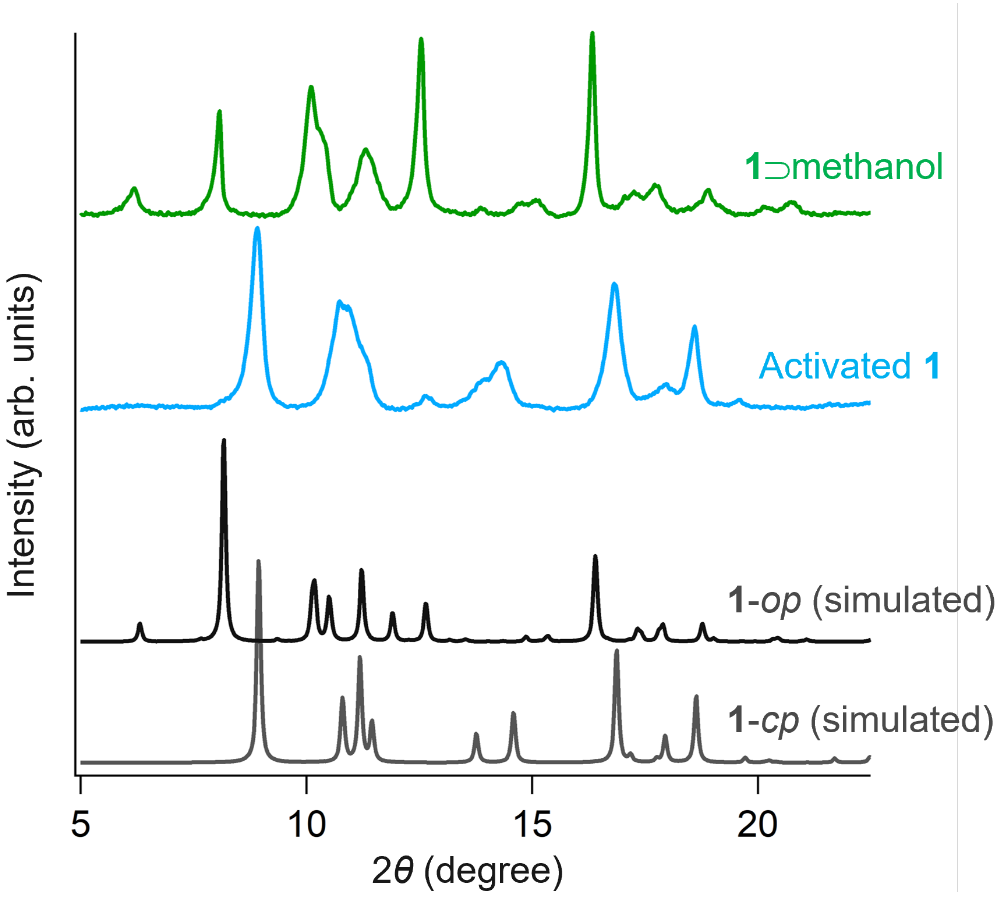


**Supplementary Fig. 1.**

PXRD patterns of activated **1** (closed phase) and **1** containing methanol (**1**⊃methanol) (open phase) and simulated pattens of **1**-*op* and **1**-*cp* phases.

By synthesizing $[Cu_2(bdc)_2(bpy)]_n$ without modulator (acetic acid), $[Cu_2(bdc)_2(bpy)]_n$ with much smaller particle size (**1’**) was obtained. Due to the shape-memory effect of small-sized $[Cu_2(bdc)_2(bpy)]_n$ (< 300 nm),[1] even after activation at 130 °C, **1’** partly showed diffraction peaks of **1’**-*op*, which is kinetically favored metastable phase and additional annealing at 200 °C gave fully closed **1’** (Supplementary Fig. 2).

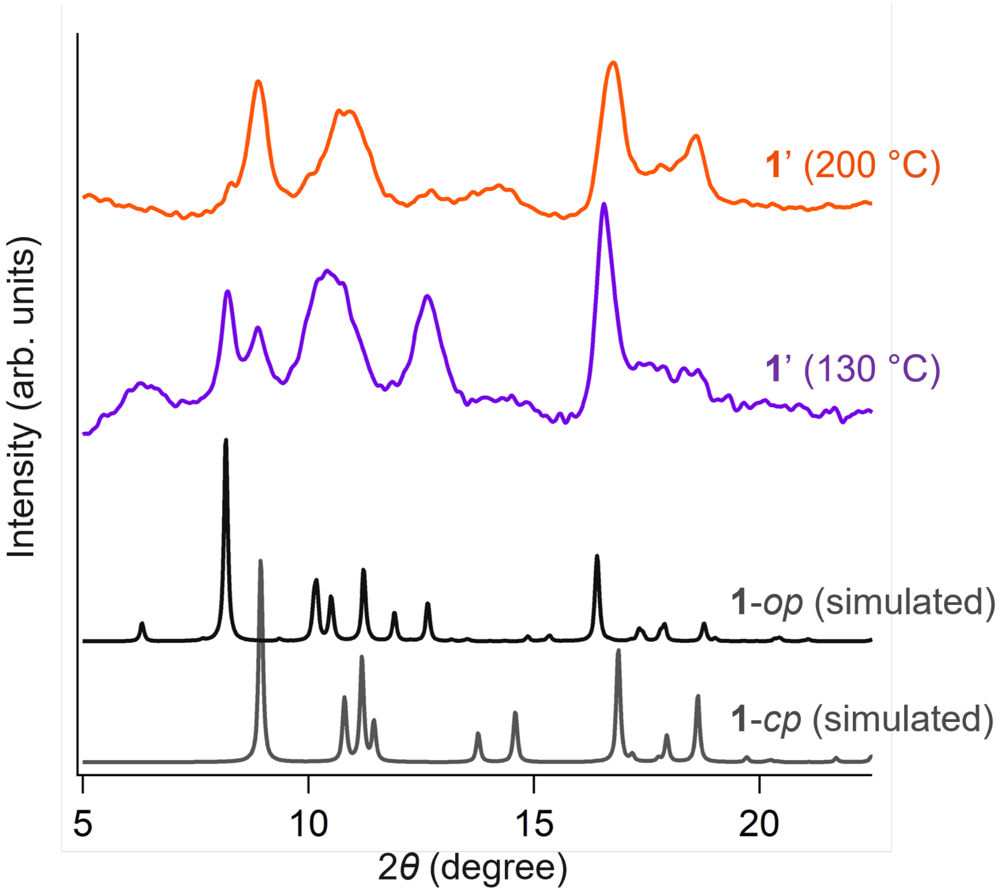


**Supplementary Fig. 2.**

PXRD patterns of small-sized **1** (**1’**). Due to the shape-memory effect of **1’**, open phase was partially remained even after the drying at 130 °C in vacuo (purple). Additional thermal annealing at 200 °C gave pure **1’**-*cp* (orange). Black and gray lines denote the simulated patterns of **1**-*op* and **1**-*cp*, respectively.

## 2. Investigation of the MOF crystal size and size comparison between MOF and PEO

Size comparison between **1** crystal and the contour length of each PEO chain is shown in Supplementary Fig. 3. Contour lengths of **PEO2k** (~15 nm) and **PEO10k** (~75 nm) are shorter than the short axis (thickness direction) of the crystal. The short axis corresponds to the bpy ligand axis, i.e., the channel direction of **1**. **PEO200k** (~1.5 μm) and **PEO4M** (~30 μm) are sufficiently longer than the thickness of **1** crystal, thus they are expected to penetrate single **1** crystal.

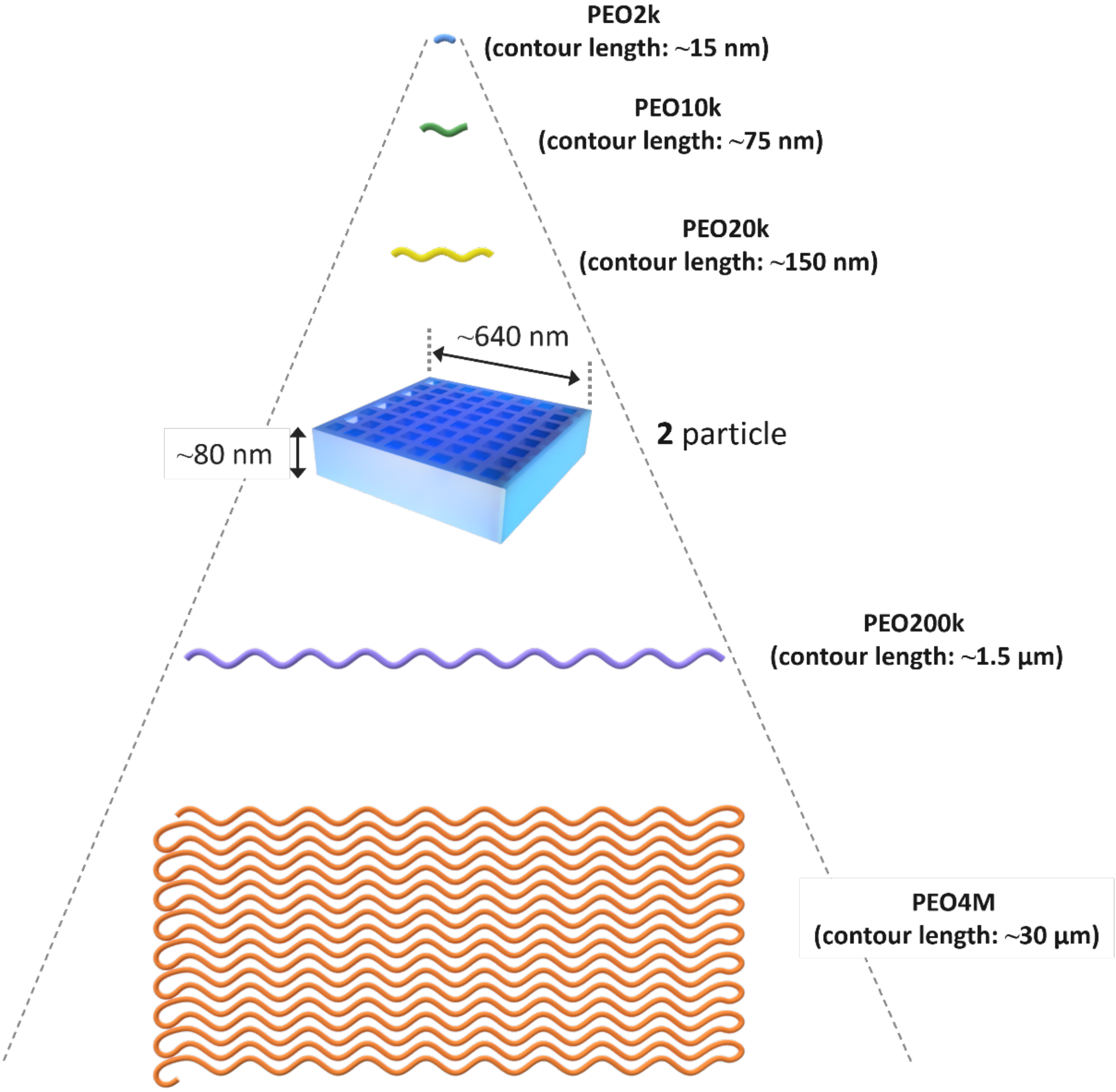


**Supplementary Fig. 3.**
Schematic illustration of the size comparison between **1** particle and PEO chains.

Statistical analysis on AFM images of **1’** showed that the mean width and thickness are 208 nm and 16 nm, respectively (Supplementally Fig. 4).

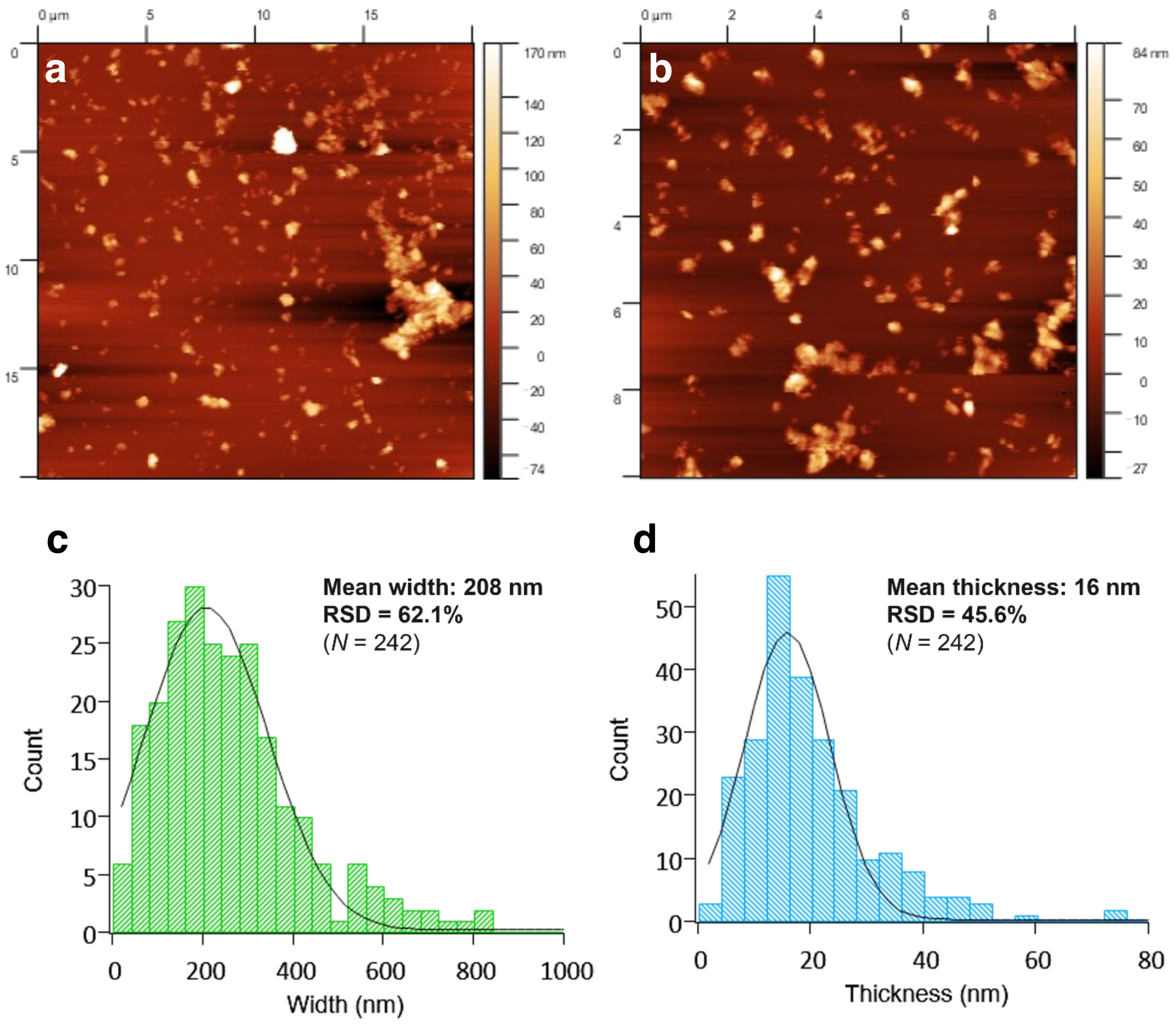


**Supplementary Fig. 4.**

**a**,**b**, Representative AFM images of the particles of synthesized **1’**. **c**,**d**, Distribution of (**c**) the diameter and (d) thickness of **1’** particles, obtained from the AFM images.

### 3. Thermogravimetric analysis of guest included 1

Supplementary Fig. 5 shows thermogravimetric (TG) curve for **1**⊃DMF. The weight loss observed until the temperature reaches 200 °C corresponds to the loss of DMF guest molecules. Based on the weight loss, the full capacity of **1** and **1’** was calculated to be 0.23 g/g and 0.18 g/g, respectively. These values are in good agreement with the previously reported value[1].

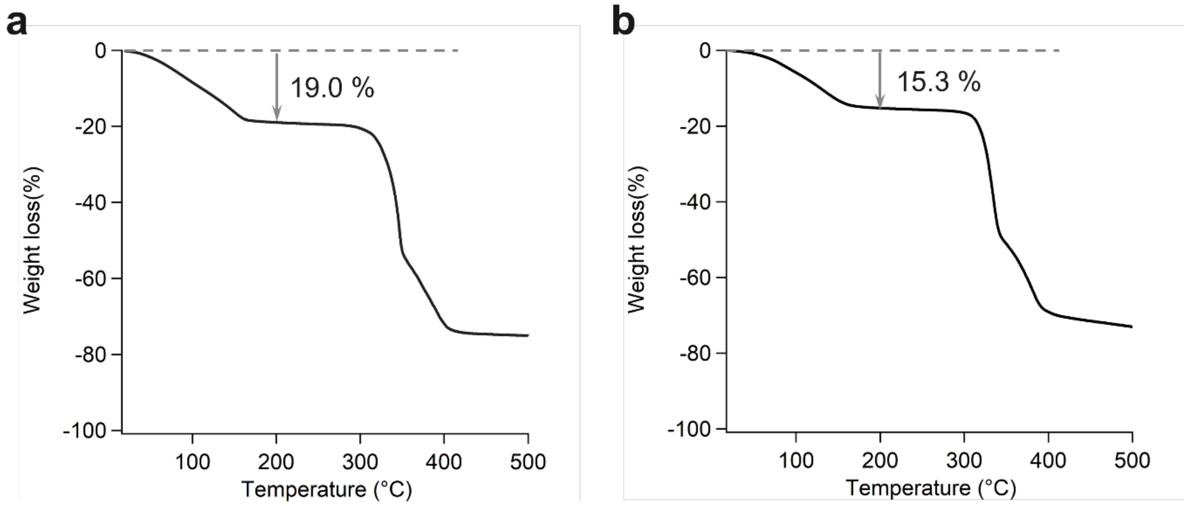


**Supplementary Fig. 5.**
TG profile of DMF included (a) **1** (**1**⊃DMF) and (b) **1’**(**1’**⊃DMF).

### 4. Discussion about the driving force of PEO penetration in 1

To investigate the thermodynamic background of PEO threading event, we measured a heat flow during the insertion process by DSC analysis. Since **1** shows structural change upon PEO insertion, we need to consider the *cp*-to-*op* deformation enthalpy ($\Delta H_{def}$) of **1** in addition to the adsorption enthalpy of PEO inclusion ($\Delta H_{ads}$). Hence, the observable DSC heat flow corresponds to $\Delta H_{def} + \Delta H_{ads}$. To have $\Delta H_{ads}$ value, which can be the main driving force of the threading event, we need to measure $\Delta H_{def}$ individually. To this end, we performed the following experiments using nano-sized **1** crystals (~210 nm in diameter), hereafter termed **1'**, as the reference material that shows the structure deformation without guest adsorption.[1]

The 210 nm-size $[Cu_2(bdc)_2(bpy)]_n$ (**1'**) was synthesized according to the literature procedure with slight modifications.[1] Due to the shape-memory effect by crystal downsizing, **1'** keeps open phase even after removal of guest molecules at room temperature.[1] The metastable open phase (**1'**-*op*) spontaneously changes to the stable closed phase (**1'**-*cp*) when heated above 200 °C. This shape-memory effect is observed for **1** whose crystal size is below approximately 300 nm.[1] Using **1'** as the reference crystal, it is possible to estimate the enthalpy change of *cp*-to-*op* phase transformation without undergoing guest adsorption/desorption process. We synthesized **1'** containing $CH_2Cl_2$ as the guest solvent. Evacuating the **1'** nanocrystals at 100 °C resulted in the mixture of metastable (**1'**-*op*) and the stable (**1'**-*cp*) phases (**1'**-*cp*/*op*) (Supplementary Fig. 6). We note that the product becomes the mixture of close and open phases (**1'**-*cp*/*op*) since the sample of **1'** contains particles larger than 300 nm due to its original particle size distribution (Supplementary Fig. 4).

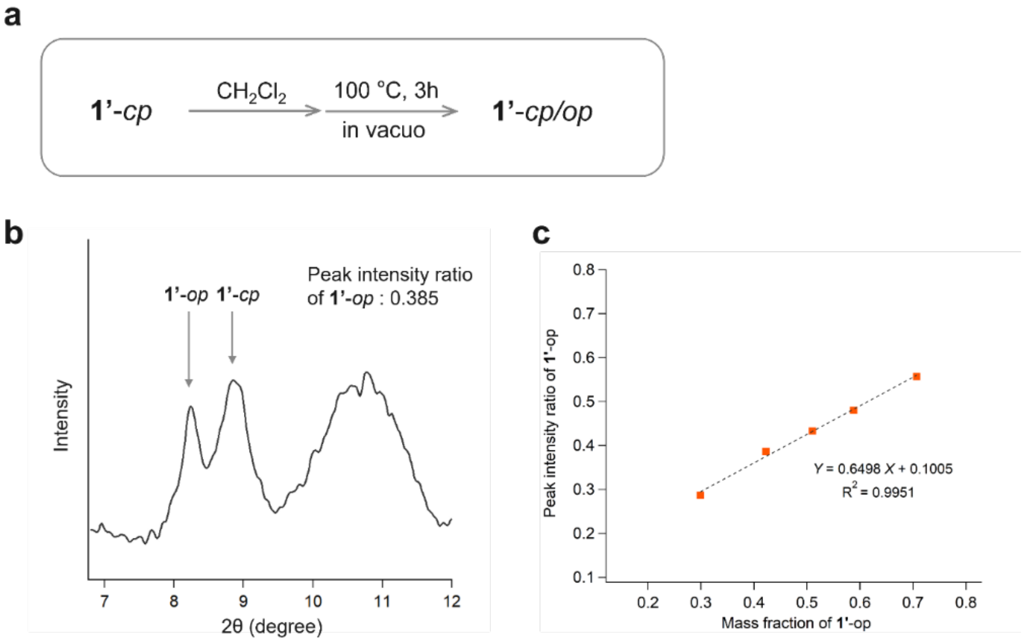


**Supplementary Fig. 6.**
**a**, **1'**-*cp* was immersed in $CH_2Cl_2$ and evacuated at 100 °C for 3 h, which gave the mixture of closed and open phases of **1'** (**1'**-*cp*/*op*). **b**, PXRD pattern of **1'**-*cp*/*op*. The peak intensity ratio of **1'**-*op* was 0.385. **c**, A relationship between PXRD peak intensity ratio of **1'**-*op* and mass fraction of **1'**-*op* for physical mixtures of **1'**-*op* and **1'**-*cp* with various mixing ratio.

To estimate phase transition enthalpy of **1'**, it is necessary to know the mass fraction of **1'**-*op* in the mixture (**1'**-*cp*/*op*). For this, we created a calibration curve using PXRD patterns of

the mixtures which were prepared by purposely mixing pure **1'**-*op* and **1'**-*cp* with various weight ratios. The intensity ratio between **1'**-*op* peak at $2\theta = 8.2°$ and **1'**-*cp* peak at 9.0° on the PXRD data was plotted as a function of the mass fraction of **1'**-*op*. For this analysis, we used the peaks at 8.2° and 9.0° as the indices of *op* and *cp* phases of **1'**, respectively, instead of the peaks at 16.4° and 16.9° (Figure 3). This is because the latter peaks showed severe overlapping due to the peak broadening caused by the small crystalline size. The calibration curve thus obtained showed a proportional trend (Supplementary Fig. 6c). Using this calibration curve, the mass fraction of **1'**-*op* in the actual mixture (**1'**-*cp*/*op*) (Supplementary Fig. 6b) was calculated to be 0.44.

In the DSC heating curve of **1'**-*cp*/*op*, an exothermic peak was observed at 150–200 °C, which corresponds to the transition from **1'**-*op* to **1'**-*cp* (Supplementary Fig. 7a). This indicates that the *op*-to-*cp* backward transition is an endothermic process. By integrating the differential curve (Supplementary Fig. 7b) between the two DSC profiles of **1'**-*cp*/*op* and **1'**-*cp* in Supplementary Fig. 7a, the total heat released during the structure change from **1'**-*cp*/*op* to **1'**-*cp* was calculated to be 46 J/g. Therefore, considering the mass fraction of **1'**-*op* in **1'**-*cp*/*op* determined above, the actual enthalpy for the transition from **1'**-*cp* to **1'**-*op* ($\Delta H_{def}$) was estimated to be 105 J/g.

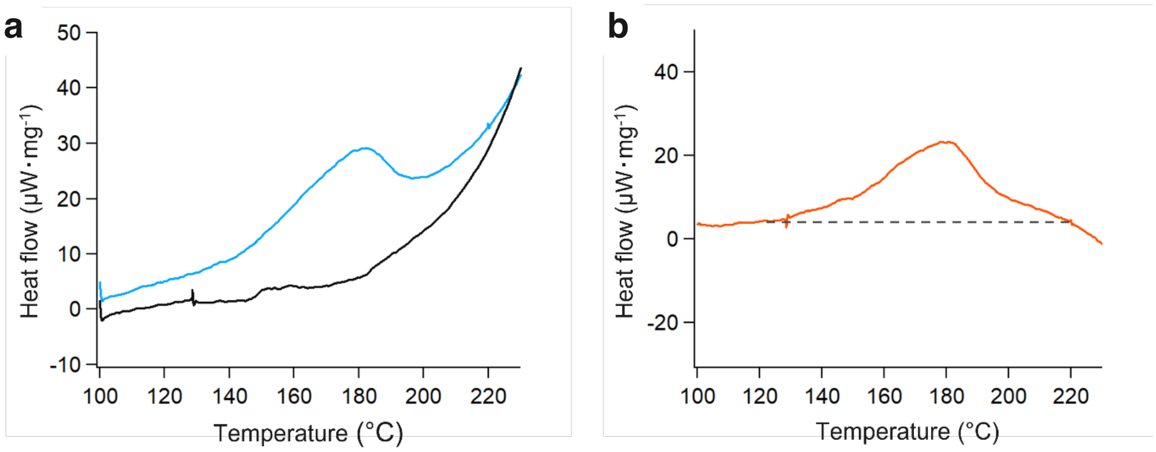


**Supplementary Fig. 7.**
**a**, DSC heating curves of **1'**-*cp* (black) and **1'**-*cp*/*op* (blue). Scan rate: 1 °C/min. Exothermic peak in **1'**-*cp*/*op* corresponds to the phase transition from **1'**-*op* to **1'**-*cp*. **b**, Subtracted curve obtained by subtracting the black curve from the blue curve in the panel **a**. The dotted line corresponds to the baseline for the integration analysis.

The PEO insertion into **1** accompanies the endothermic structure deformation process ($\Delta H_{def}$) and exothermic PEO adsorption process ($\Delta H_{ads}$). Finally, we measured DSC heat flow of the PEO insertion into **1**, which shows *cp*-to-*op* structure change, using **PEO2k** as the guest. A mixture of **PEO2k** (4.02 mg) and **1** (2.88 mg) was placed in an Al pan and subjected to the DSC measurement (20 °C–100 °C, 1 °C/min). DSC peak was observed at 53 °C at which melting of **PEO2k** and the infiltration occurred simultaneously, releasing the heat ($\Delta H_{obs}$) of 147 J per gram of PEO in total (Supplementary Fig. 8). As the bulk **PEO2k** melted at this temperature, the enthalpy of fusion ($\Delta H_f$) should be also taken into account. $\Delta H_f$ was determined to be 193 J/g from the integration of the DSC heating curve of **PEO2k** alone (Supplementary Fig. 8). Therefore, $\Delta H_{ads}$ was calculated as $\Delta H_{ads} = \Delta H_{obs} - \Delta H_{def} - \Delta H_f = (147 \times 4.02) - (105 \times 2.88) - (193 \times 4.02) = -487$ mJ. Considering the maximum adsorption capacity of **1** (0.23 g/g), the actual amount of **PEO2k** adsorbed in **1** can be calculated as 2.88 × 0.23 =

0.662 mg. Therefore, $\Delta H_{ads}$ is calculated as $\Delta H_{ads} = -487 / 0.662 = -736$ J/g (per gram of adsorbed **PEO2k**), which is converted to –32 kJ/mol (per PEO repeating unit). As $\Delta H_{ads}$ is the negative value, the PEO threading is an exothermic, enthalpy driven process. The $\Delta H_{ads}$ value is larger than that observed previously for other MOF/PEO systems, e.g. –7.7 kJ/mol per repeating unit of PEO for the insertion into $[Zn_2(1,4\text{-ndc})_2(ted)]_n$ (ndc = naphthalenedicarboxylate, ted = triethylenediamine).[2] The strong affinity of **1** and PEO can be attributed to the narrow pore size of **1**. It should be noted that the kinetic factor may also give a substantial effect on the overall penetration efficiency of the ultralong guests. Please also see the discussion in Section III.2.

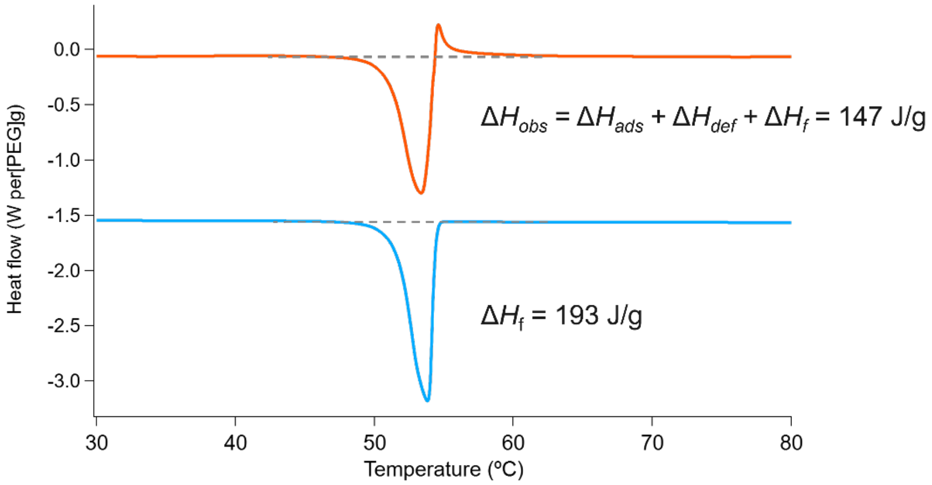


**Supplementary Fig. 8.**

DSC heating curves of **1/PEO2k** mixture (top) and **PEO2k** (bottom) in the heating rate of 1 °C/min. The broken lines correspond to the baselines for the integration. During the heating processes, the endothermic heats of 147 J and 193 J per gram of PEO were observed for **1/PEO2k** mixture and **PEO2k**, respectively.

## II. Discussion about the infiltration process of PEO into 1

### 1. In-situ PXRD analysis using end-capped PEO20k with bulky *tert*-butyldiphenylsilyl (TBDPS) group

We performed a control experiment using end-capped **PEO20k** with bulky *tert*-butyldiphenylsilyl (TBDPS) group (**PEO20k**-TBDPS). The projection diameter of TBDPS group exceeds the window size of **1**-*op*. This inhibits PEO infiltration into **1** nanopores. As is obvious from Supplementary Fig. 9, TBDPS-terminated **PEO20k** did not induce structure change of **1** during the heating of the **1**/PEO mixture. This result clearly suggests that the structure change of non-capped PEO is caused by the insertion of PEO chains into the nanopores of **1** from the termini.

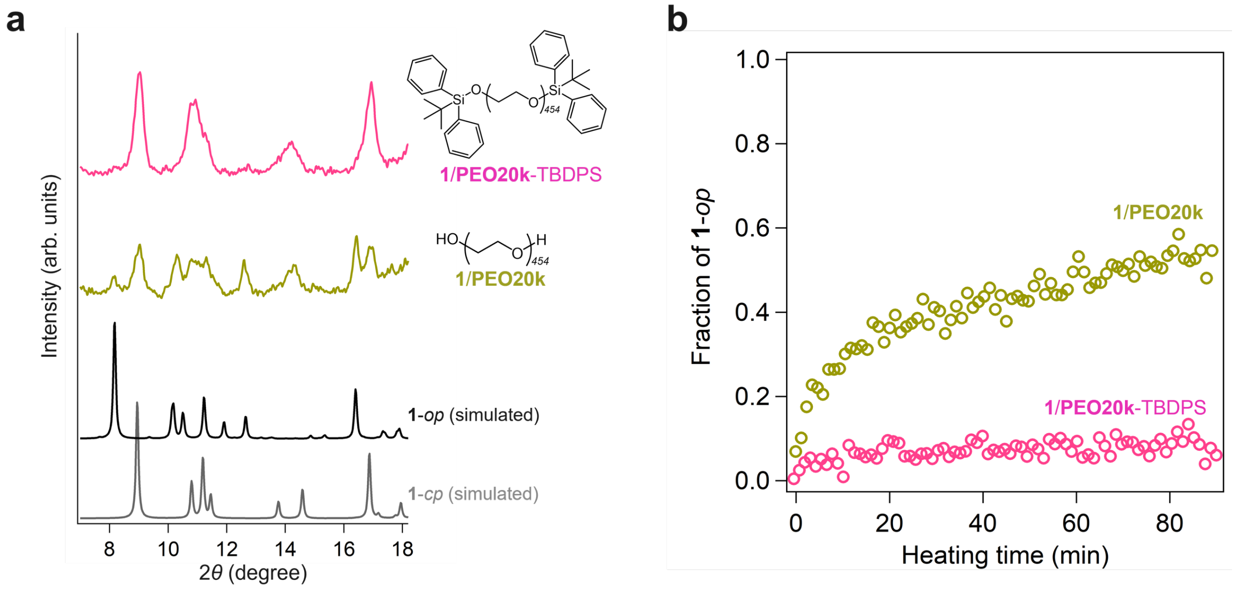


**Supplementary Fig. 9.**

**a**, PXRD profiles for the mixture of **1**/**PEO20k** (yellow) and **1**/**PEO20k**-TBDPS (pink) annealed at 120 °C for 90 min. **b**, Evolution of the fraction of **1**-*op* in contact with molten **PEO20k** (yellow) and **PEO20k**-TBDPS (pink), monitored at 120 °C.

## III. Structural investigation of polypseudoMOFaxane

### 1. PXRD patterns of 1/PEO4M composites with less PEO loading amount

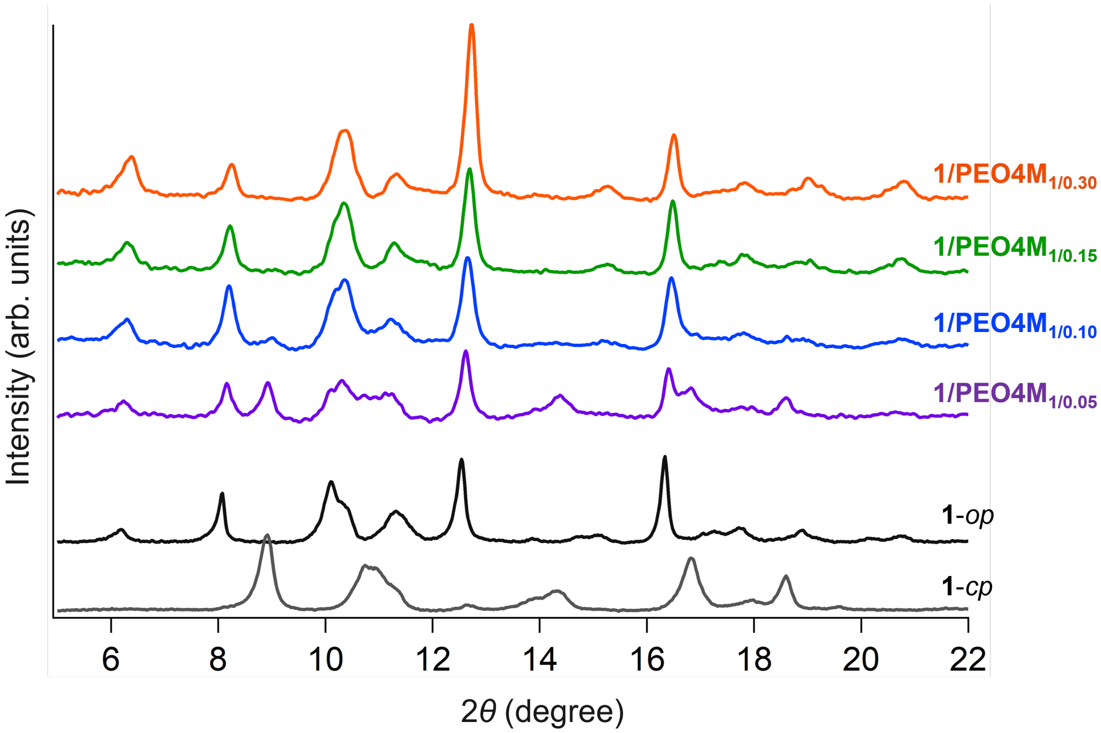


**Supplementary Fig. 10.**

PXRD profiles for the **1/PEO4M$_{1/0.30}$** (orange), **1/PEO4M$_{1/0.15}$** (green), **1/PEO4M$_{1/0.10}$** (blue) and **1/PEO4M$_{1/0.05}$** (purple). **1**-*cp* phase appeared in **1/PEO4M$_{1/0.10}$**, indicating approx. 1/0.10 ratio (wt/wt) is the critical point at which all MOF microcrystals encounter PEO chain to be penetrated with **PEO4M**. At 1/0.05 loading (**1/PEO4M$_{1/0.05}$**), the diffraction peak intensity corresponding to the **1**-*op* phase decreased. The fraction of **1**-*op* phase in the **1/PEO4M$_{1/0.05}$** composite was determined using the calibration curve given in Supplementary Fig. 6. The **1**-*op* fraction was 81.5% even at 1/0.05 **PEO4M** loading. This high gate-opening efficacy of **PEO4M** clearly underpins the formation of the polythreading configuration.

## 2. Estimation of the coverage of polypseudoMOFaxane

The percent coverage of PEO with **1** was estimated based on the PEO loading amount and experimental gas adsorption analysis. Firstly, we calculated the number of pore entrances presenting on the crystal surfaces. Based on the single crystal structure of **1** and the mean particle dimension (640 nm × 640 nm × 80 nm), the number of pore entrances on the crystal surfaces was calculated to be $3.5 \times 10^5$/particle. We assume that 100% coverage of PEO with **1** can be achieved at the point of the maximum loading capacity of PEO in **1** (0.23 g/g). In other words, all pores (i.e. pore entrances) of **1** are threaded by PEO chains at >0.23 g/g PEO loading. Under this assumption, the coverage can be simply expressed as $0.23/x$ where $x$ is the weight ratio of PEO to that of **1**. For example, the coverage of the **1/PEO4M$_{1/0.3}$** composite ($x$ = 0.30) can be 0.23/0.30 = 0.77 (77%). In reality, however, it is unlikely that PEO chains penetrate all pores of **1** microcrystals due to the kinetic reason attributed to the ultralong length and entangled conformation. Indeed, the $N_2$ gas adsorption analysis showed that the **1/PEO4M$_{1/0.3}$** composite still has effective microporosity (Supplementary Fig. 11). **1** microcrystals in the **1/PEO4M$_{1/0.3}$** composite showed the adsorption capacity of ~68 mL ($P/P_0$ = 0.9) that is approximately 33% of that for the pristine **1** in open form (~205 mL). In other words, 67% of the MOF pores (i.e. pore entrances) are involved in threading **PEO4M** in the composite. Based on this result, the actual coverage of the **1/PEO4M$_{1/0.3}$** composite can be estimated as (0.23/0.30) × 0.67 = 0.51 (51%). By following this estimation method and the assumptions, the coverage of **1/PEO4M$_{1/1}$** is also calculated to be 15%.

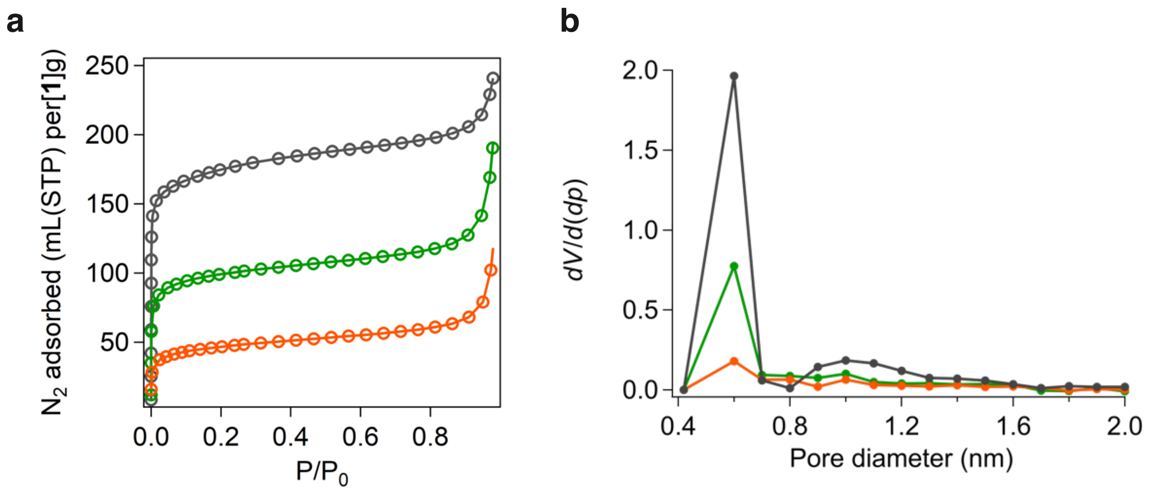


**Supplementary Fig. 11.**

**a**, $N_2$ adsorption isotherms of the pristine **1** (gray), **1/PEO4M$_{1/0.15}$** (green), and **1/PEO4M$_{1/0.3}$** (orange), measured at 77 K. **b**, Pore size distribution of the pristine **1** (gray), **1/PEO4M$_{1/0.15}$** (green), and **1/PEO4M$_{1/0.3}$** (orange), calculated by MP (micropore) method using the $N_2$ adsorption isotherms shown in the panel **a**. It was observed that the adsorption capacity decreases with increasing the PEO loading amount while the mean pore size is not significantly changed. This indicates that the decrease of adsorption capacity is ascribed to the decrease in the number of vacant pores by PEO threading.

### 3. Particle size distribution analysis of polypseudoMOFaxane

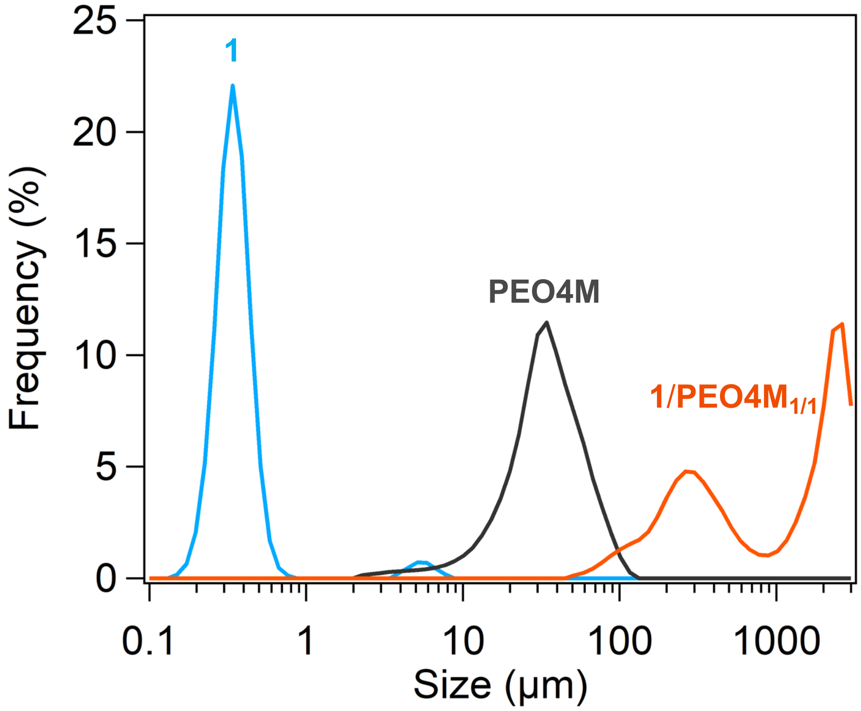


**Supplementary Fig. 12.**

Particle size distribution data for **1** (blue), **PEO4M** (gray), and **1/PEO4M$_{1/1}$** (orange) composite dispersed in $CHCl_3$ at room temperature. The samples were dispersed by stirring for 5 min and subjected to the laser-scattering particle size distribution measurements. The **1** particle alone and **PEO4M** solution showed the monodisperse peak at the size of ~0.34 μm and ~34 μm, respectively. On the other hand, **1/PEO4M$_{1/1}$** composite showed the presence of much larger particles that are attributed to the formation of poly-threaded complex.

## 4. AFM imaging of polypseudoMOFaxane

The AFM imaging for **1**, **1/PEO4M$_{1/0.3}$**, and **1/PEO4M$_{1/1}$** were performed as follows. Each sample was dispersed by stirring for 5 min (25 °C) in DMF or chloroform (1 mg/mL) and deposited on a mica substrate by spin coating (2500 rpm, 5 sec). The deposited particles were imaged using Asylum Research model MFP-3D Origin operated in non-contact tapping mode. A silicon cantilever (OMCL-AC240TS, Olympus) with a spring constant ranging from 0.6 to 3.5 N/m (resonant frequency of 50-90 kHz) was used and calibrated by the thermal fluctuation method. Igor Pro software (WaveMetrics) was used for all of the data acquisition and analysis.

While the AFM image of the pristine **1** showed individually dispersed microcrystals (Fig. 2a), the **1/PEO4M$_{1/1}$** composite showed obvious agglomerations of the crystals (Fig. 5). In the **1/PEO4M$_{1/1}$** composite, the microcrystals of **1** form loosely tethered each other to form gatherings, rather than forming massive aggregates. This agglomeration formation of **1/PEO4M$_{1/1}$** is consistent with the results of the particle-size distribution measurements (Supplementary Fig. 12). This morphological feature is in good agreement with what we envisioned for the polypseudoMOFaxane (Fig. 1d) in which polymer chains are weaving and tethering multiple MOF particles, forming the loose network structure. This morphology is also reasonable when considering the PEO coverage of the **1/PEO4M$_{1/1}$** composite, which is estimated to be 15% (see Section III.2). It should be noted that the most of **PEO4M** chains were observed in the background as a thin film homogeneously covering the substrate since the amount of **PEO4M** is in an excess to the MOF capacity in this 1/1 composite.

Interestingly, the 1/0.3 composite, **1/PEO4M$_{1/0.3}$**, showed more intuitive morphology supporting the polypseudoMOFaxane structure (Supplementary Fig. 13). For the **1/PEO4M$_{1/0.3}$** composite, dendritic PEO crystals were observed at the periphery of each particle. This intriguing morphology that all PEO radial domains are localized in contact with **1** microcrystals, and neither individual PEO crystals nor MOF particles were observed. This morphological feature supports the polypseudoMOFaxane structure in which extremely long PEO chains topologically bind multiple **1** microcrystals. For comparison, we performed AFM imaging for an instant mixture of **1** and **PEO4M**. **1** was dispersed in chloroform (1 mg/mL) by sonication for 20 min prior to mixing. The **1** suspension and **PEO4M** solution (1 mg/mL in chloroform) were mixed by stirring for 5 min (25 °C) to have the instant mixture in 1/1, wt/wt, ratio. The mixture was deposited on a mica substrate by spin coating (2500 rpm, 5 sec). The AFM image of the mixture showed slight agglomerations of **1** microcrystals, but with different morphology (Supplementary Fig. 14). No localization of PEO chains was observed. The PEO chains appeared as the homogeneous background with uniformly distributed dot-like nanocrystals. These observations suggest that the polypseudoMOFaxane structure is not effectively formed just by instant mixing in solution phase. To obtain the polythreading structure, successive evaporation of the sacrificial guest solvent (chloroform) at high temperature is indispensable process due to the slow diffusion of PEO in **1**.

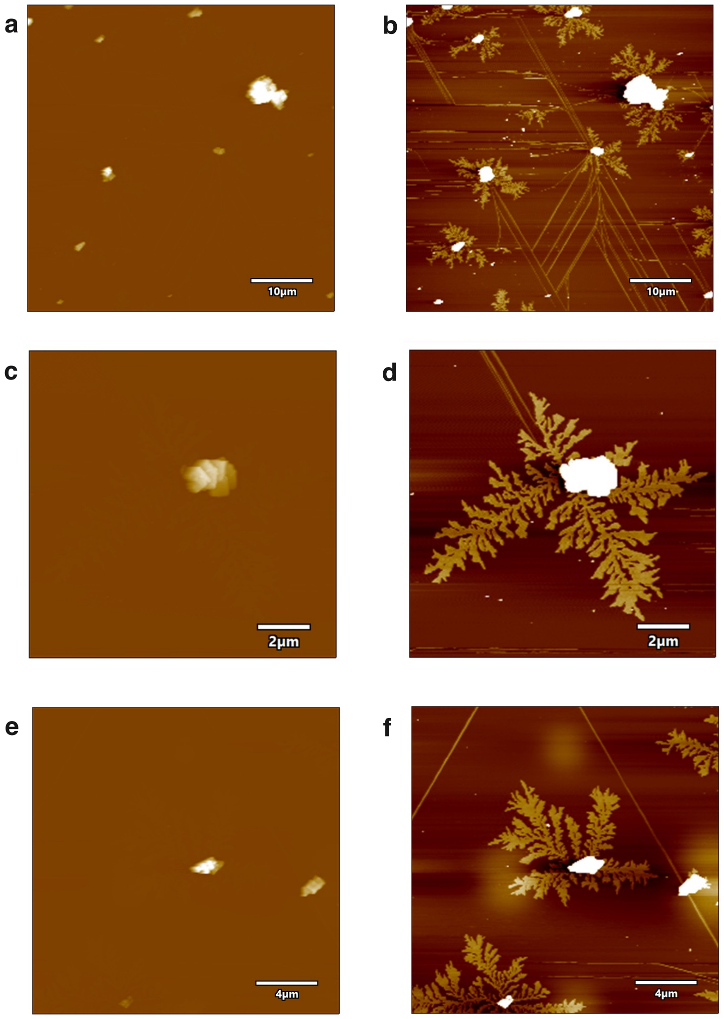


**Supplementary Fig. 13.**

Topographic AFM images of **1/PEO4M$_{\mathbf{1/0.3}}$** composite deposited on a mica substrate. **a,b,c,** The AFM images highlighting separated agglomerates, and **d,e,f,** those in different height contrast, respectively. The contrast is adjusted to visualize the radial PEO crystals surrounding each agglomerate.

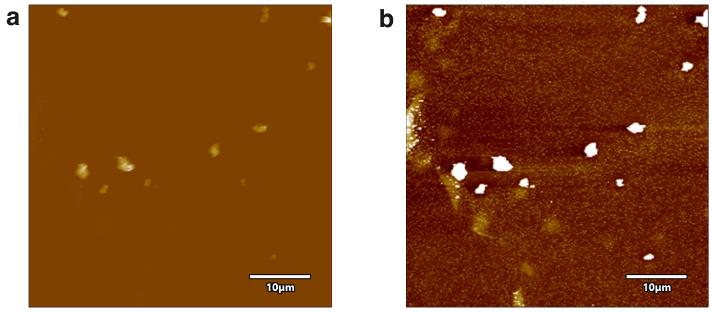


**Supplementary Fig. 14.**

Topographic AFM images of the **1** and **PEO4M** mixture (1/1, wt/wt) deposited on a mica substrate. **a,** The AFM image highlighting separated agglomerates, and **b,** that in different height contrast. The contrast is adjusted to visualize the location of PEO domains. PEO chains were observed in the background as uniformly distributed dot-like nanocrystals.

## IV. Effect of polyMOFaxane structure on the properties of 1/PEO composites

### 1. Investigation of the effect of the polypseudoMOFaxane structure on the crystallization rate of PEO

**Friedman plot.** In this study, to investigate crystallization behavior of PEO in **1**/PEO composites we measured their crystallization exotherm by DSC analyses using various cooling rates of 0.4, 0.6, 1, 2, and 5 °C/min (Supplementary Fig. 15). The crystallization kinetics was calculated based on the crystallization exotherms plotted as a function of temperature *T*. Under the assumption that the evolution of crystallinity is proportional to the heat released during crystallization, the relative degree of crystallinity, $\alpha$, is calculated as,

$$\alpha(T) = \frac{\int_{T_0}^{T} (dH/dT) dT}{\int_{T_0}^{T_\infty} (dH/dT)\, dT}, \tag{1}$$

where *dH* denotes the measured crystallization enthalpy during an infinitesimal temperature interval $dT$. $T_0$ and $T_\infty$ are the temperatures at which the crystallization initiates and completes, respectively (Supplementary Fig. 16). Friedman[2] and Vyazovskin[3] developed a differential isoconversional methods for calculating the effective activation energy, *E*, for melt crystallization process based on Eq. 2,

$$\ln\left(\frac{d\alpha}{dt}\right) = \ln\left(\beta \frac{d\alpha}{dT}\right) = \ln A + \ln f(\alpha) - \frac{E}{RT}, \tag{2}$$

where $\beta$ is the cooling rate and $f(\alpha)$ is the function describing the reaction mechanism. *R* is the universal gas constant and *A* is a pre-exponential factor. By plotting ln($d\alpha/dt$) as a function of 1/*T*, a straight line with the slope equal to –*E*/*R* was obtained for each sample at respective $\alpha$ (Supplementary Fig. 17). *E* at given α can be calculated from the slope. We estimated *E* for pristine PEOs (**PEO2k** and **PEO4M**,) and the **1**/PEO composites (**1/PEO2k$_{1/1}$** and **1/PEO4M$_{1/1}$**) at α of 10, 30, 50, 70% (Supplementary Table 1). To facilitate the discussion of the PEO crystallization behavior, the data of Supplementary Fig. 16 and 17 were reorganized and replotted in Supplementary Fig. 18 and 19, respectively.

In the DSC cooling curves (Supplementary Fig. 15) and the temperature dependence of $\alpha$ (Supplementary Fig. 18), it was observed that the crystallization of the **1**/PEO composite starts earlier than that of the respective pristine PEOs. This resulted in the higher (~1 °C) crystallization temperature of the composites (Supplementary Fig. 15). In the case of **PEG2k** and **1/PEG2k$_{1/1}$**, the PEO crystallization proceeded rapidly with making similar trend of the crystallization curves in all cooling rates (Supplementary Fig. 18a,c). On the other hand, in the crystallization curves for **PEO4M** and **1/PEG4M$_{1/1}$**, the trend was largely different. The crystallization of **1/PEG4M$_{1/1}$** was significantly retarded as the crystallization proceeds (Supplementary Fig. 18b,d) while the pristine **PEO4M** showed the normal crystallization trend that is similar to that of **PEG2k**. We ascribe this significant retardation of the PEO crystallization to the penetrating structure in which each PEO chain is partially trapped by **1** microcrystals via topological constraints.

In Supplementary Fig. 19, the trends observed for the pristine **PEO2k** and the corresponding composite looks similar (Supplementary Fig. 19a,b) while the pronounced difference was observed for **PEO4M** and its composite (Supplementary Fig. 19c,d). The change in the slope displayed in the figures corresponds to the change in the activation energy of crystallization at the respective degree of crystallization, $\alpha$. This represents the effect of penetration structure in polypseudoMOFaxane (Fig. 6).

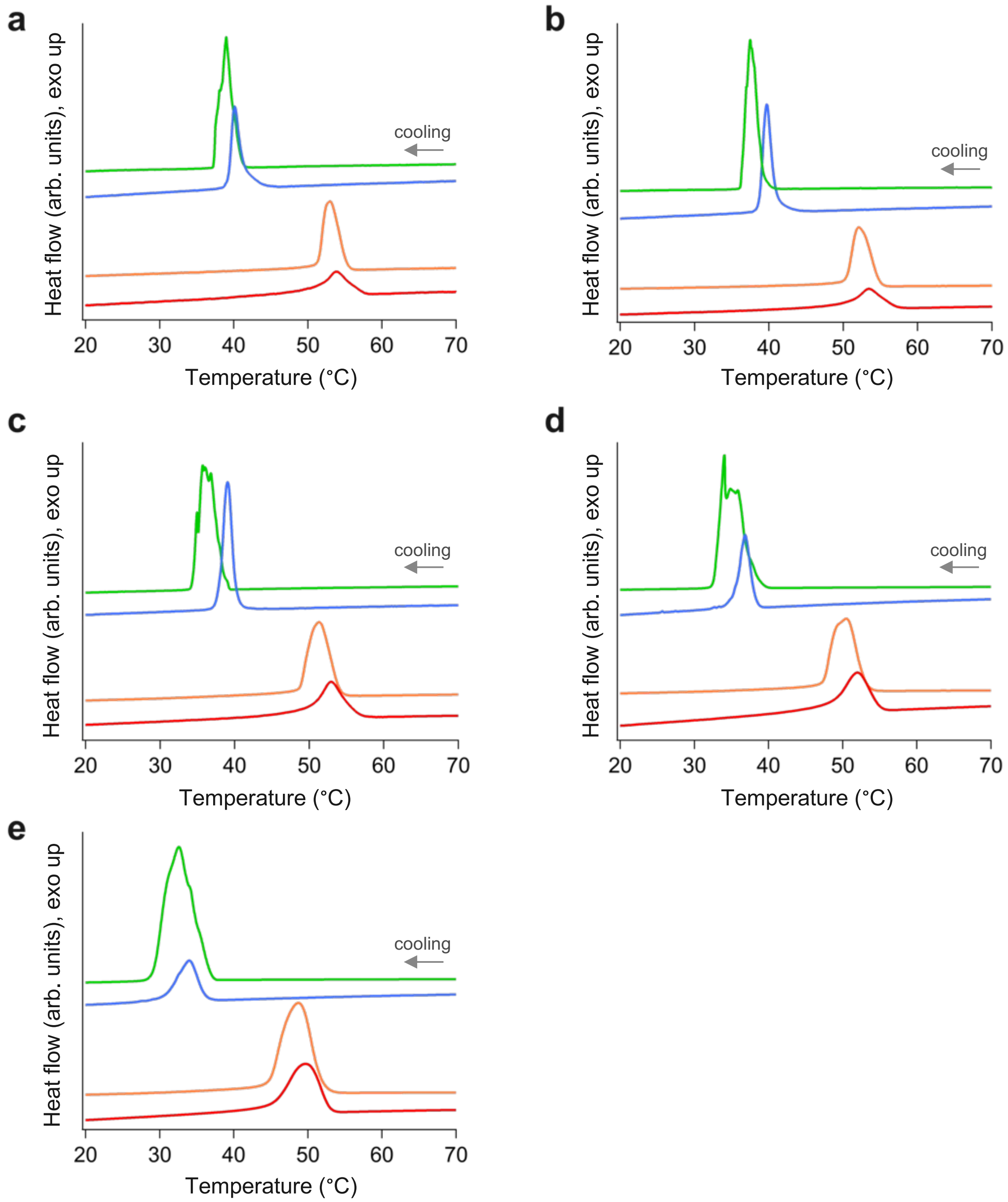


**Supplementary Fig. 15.**

**a**–**e**, DSC cooling curves of **PEO2k** (green), **1**/**PEO2k$_{1/1}$** (blue), **PEO4M** (orange), and **1**/**PEO4M$_{1/1}$** (red) recorded at the cooling rate of 0.4 °C/min (**a**), 0.6 °C/min (**b**), 1 °C/min (**c**), 2 °C/min (d), and 5 °C/min (**e**).

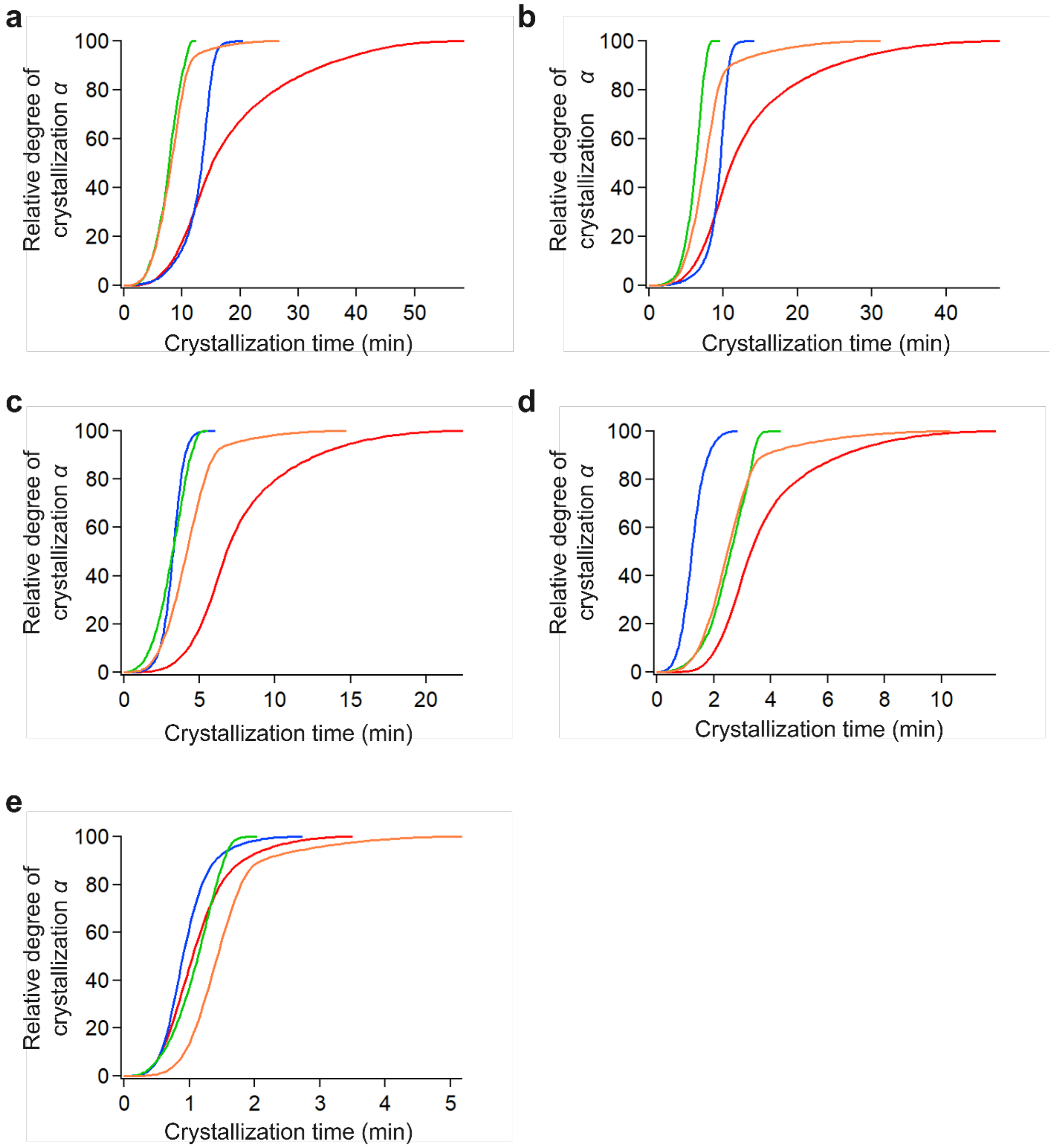


**Supplementary Fig. 16.**

**a–e**, Evolution of relative degree of crystallization, $\alpha$, of **PEO2k** (green), **1**/**PEO2k$_{1/1}$** (blue), **PEO4M** (orange), and **1**/**PEO4M$_{1/1}$** (red) as a function of the crystallization time at the cooling rate of 0.4 °C/min (**a**), 0.6 °C/min (**b**), 1 °C/min (**c**), 2 °C/min (**d**), and 5 °C/min (**e**).

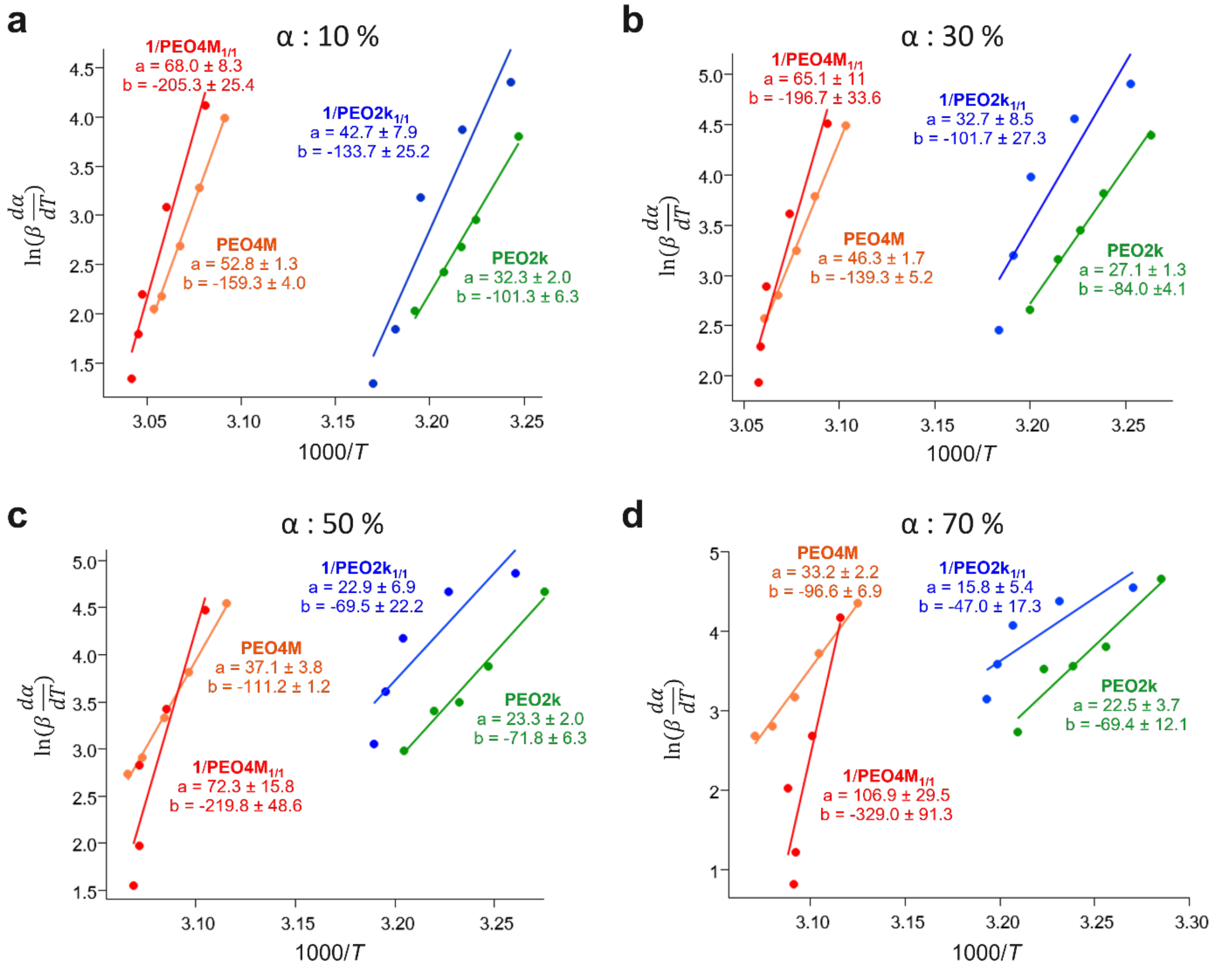


**Supplementary Fig. 17.**

**a**–**d**, The Friedman plots based on Eq. 2 for **PEO2k** (green), **1/PEO2k$_{1/1}$** (blue), **PEO4M** (orange), and **1/PEO4M$_{1/1}$** (red) at $\alpha$ of 10% (**a**), 30% (**b**), 50% (**c**), and 70% (**d**). The slope and the intercept values of the linear fitting results for each plot are shown as a and b, respectively, with the standard deviation.

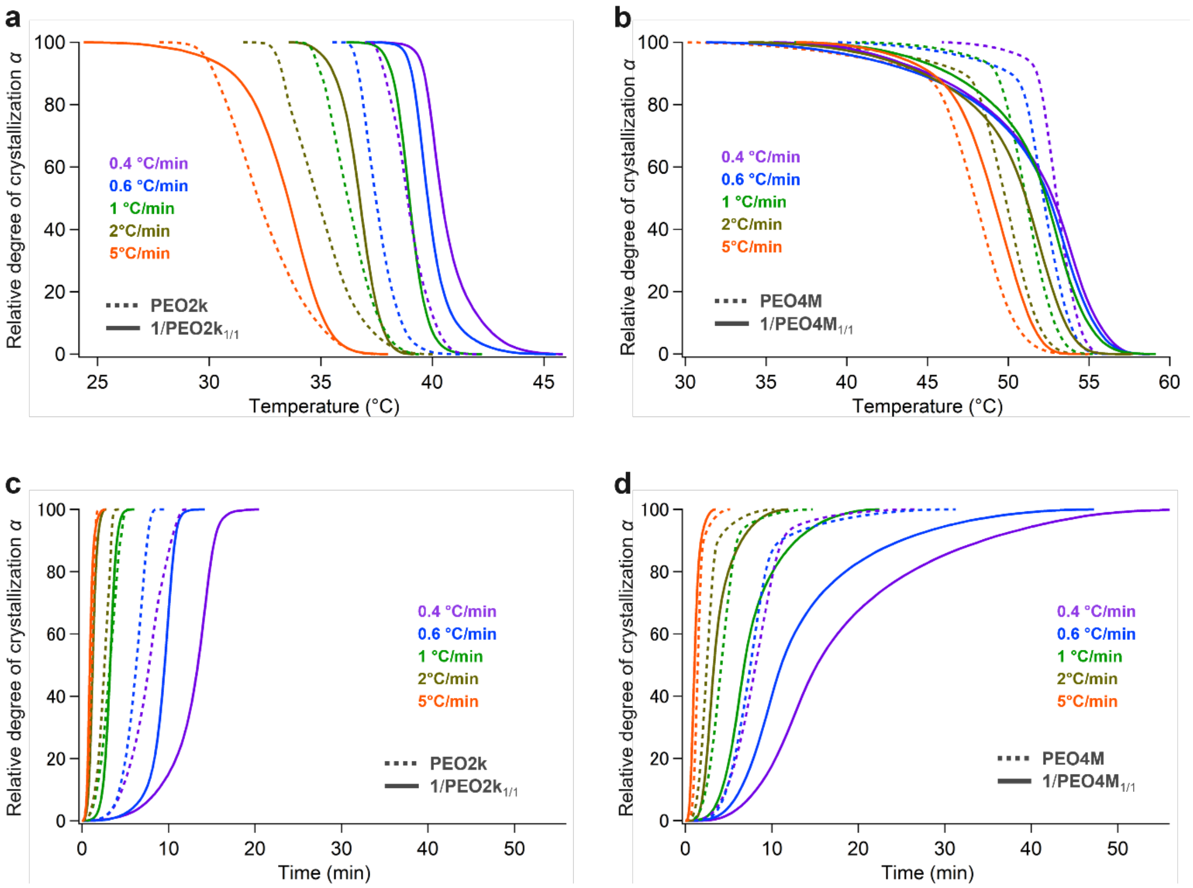


**Supplementary Fig. 18.**

**a-d,** Evolution of relative degree of crystallization, $\alpha$, of **PEO2k** (dotted lines) and **1/PEO2k$_{1/1}$** (solid lines) (**a**,**c**), and **PEO4M** (dotted lines) **1/PEO4M$_{1/1}$** (solid lines) (**b**,**d**) at cooling rate of 0.4 °C/min (purple), 0.6 °C/min (blue), 1 °C/min (green), 2 °C/min (yellow), and 5 °C/min (orange) against temperature (**a**,**b**) and crystallization time (**c**,**d**).

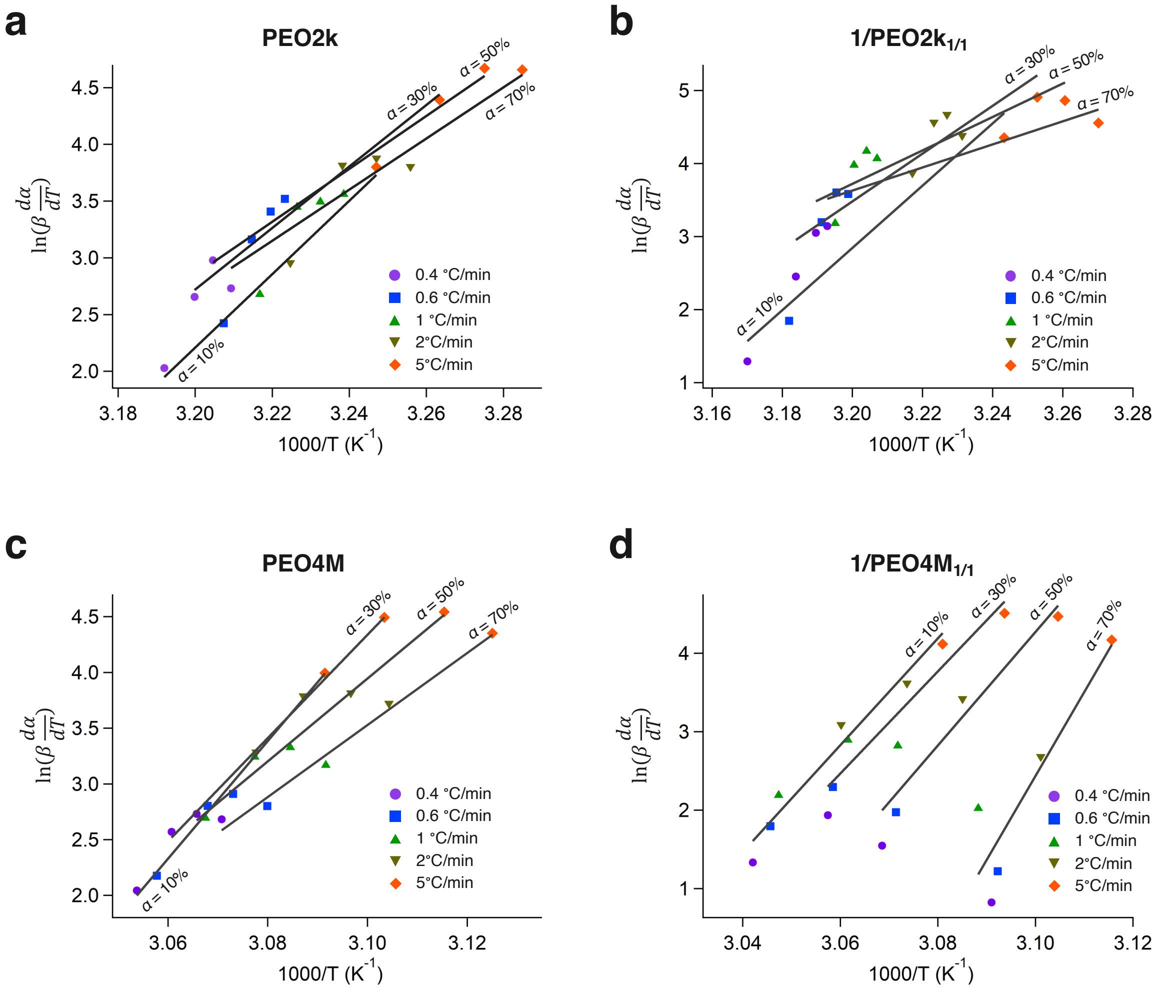


**Supplementary Fig. 19.**

**a–d,** The Friedman plots based on Eq. 2 for **PEO2k** (**a**), **$1/PEO2k_{1/1}$** (**b**), **PEO4M** (**c**), and **$1/PEO4M_{1/1}$** (**d**) at $\alpha$ of 10%, 30%, 50%, and 70%.

**Supplementary Table 1.**

The effective activation energy, $E$, (kJ/mol) of the PEO crystallization for **PEO2k**, **$1/PEO2k_{1/1}$**, **PEO4M**, and **$1/PEO4M_{1/1}$** calculated from the Friedman plot. Data are presented as mean ± SE that represents an error associated with uncertainties in the fits used to calculate the effective activation energy.

| $\alpha$(%) | **PEO2k** | **$1/PEO2k_{1/1}$** | **PEO4M** | **$1/PEO4M_{1/1}$** |
|---|---|---|---|---|
| 10 | –269 ± 16 | –355 ± 65 | –439 ± 11 | –566 ± 69 |
| 30 | –225 ± 11 | –272 ± 71 | –385 ± 14 | –542 ± 91 |
| 50 | –194 ± 16 | –190 ± 58 | –309 ± 10 | –601 ± 131 |
| 70 | –187 ± 31 | –132 ± 45 | –268 ± 19 | –889 ± 245 |

## 2. Solvent washing experiment on 1/PEO4M composite to investigate kinetic prevention of the unthreading reaction

Supplementary Fig. 20 displays PXRD patterns of the composites, **1/PEO2k$_{1/1}$** and **1/PEO4M$_{1/1}$**, before and after rigorous stirring in dichloromethane (DCM) at room temperature for 6 h. DCM is a good solvent for PEO. Both composites showed **1**-*op* phase before washing with DCM. After washing for 6 h, **1/PEO2k$_{1/1}$** showed a formation of **1**-*cp*, which indicates that a part of **PEO2k** was washed out and fell off from **1** during the washing treatment. In contrast, **1/PEO4M$_{1/1}$** showed no **1**-*cp* phase in the PXRD profile after washing.

In order to better understand the PEO-holding mechanism, we measured the amounts of PEO remaining in **1** after the 6 h-washing treatment for **1/PEO2k$_{1/1}$** and **1/PEO4M$_{1/1}$**. In addition, to determine which factor (chain entanglement or physical interaction) prevents the unthreading reaction, **1/PEO4M$_{1/0.3}$** was also examined for comparison to **1/PEO4M$_{1/1}$**. The entanglements of PEO chains in **1/PEO4M$_{1/0.3}$** are expected to be lower than those in **1/PEO4M$_{1/1}$**, as deduced from the estimated PEO coverage values: 51% for **1/PEO4M$_{1/0.3}$** and 15% for **1/PEO4M$_{1/1}$** (see Section III.2). The result showed that **1/PEO4M$_{1/0.3}$** retained only ~0.06 g/g of PEO after washing (Supplementary Fig. 21) whereas **1/PEO4M$_{1/1}$** retained 0.25 g/g after the same treatment. This suggests that preventing the unthreading reaction is mainly due to the chain entanglements, rather than the physical interactions between **1** and PEO.

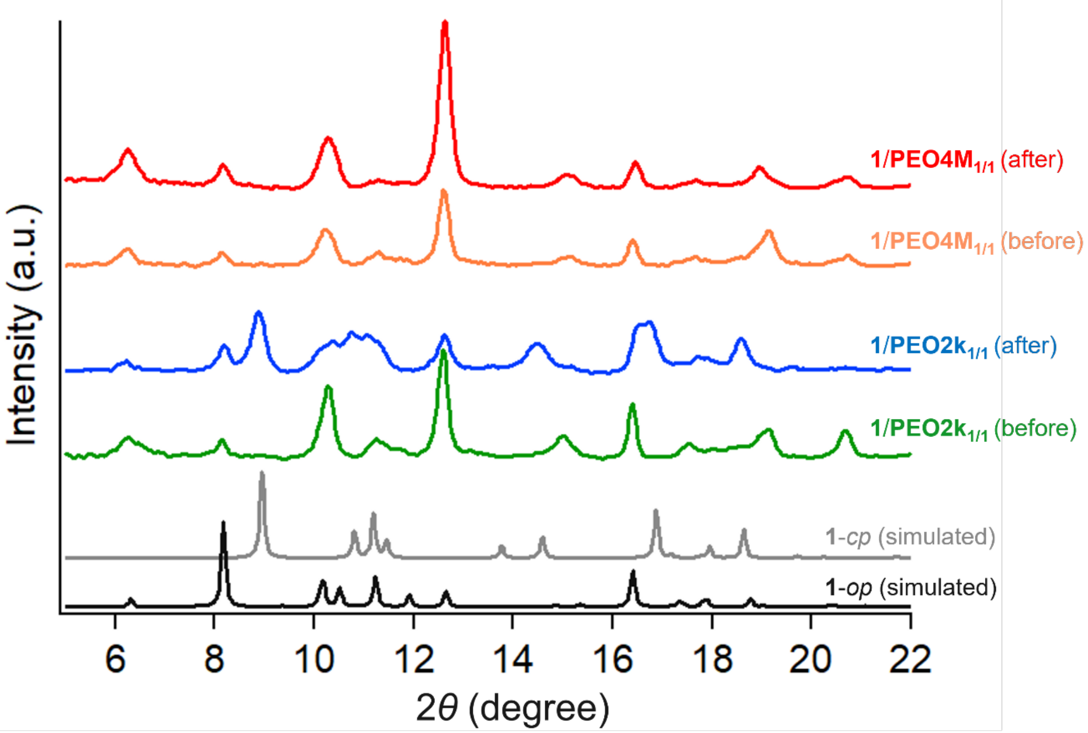


**Supplementary Fig. 20.**

PXRD patterns of **1/PEO2k$_{1/1}$** and **1/PEO4M$_{1/1}$** before and after the solvent washing with DCM for 6 h.

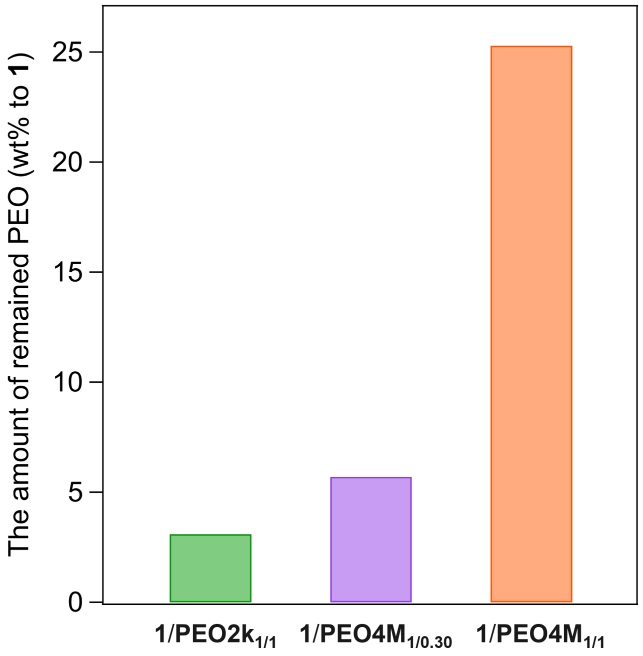


**Supplementary Fig. 21.**

The amount of PEO in the composites **1/PEO2k$_{1/1}$**, **1/PEO4M$_{1/0.3}$**, and **1/PEO4M$_{1/1}$** after the DCM-washing for 6 h. The amount of PEO was determined by $^{1}$H NMR analysis on the digested sample.

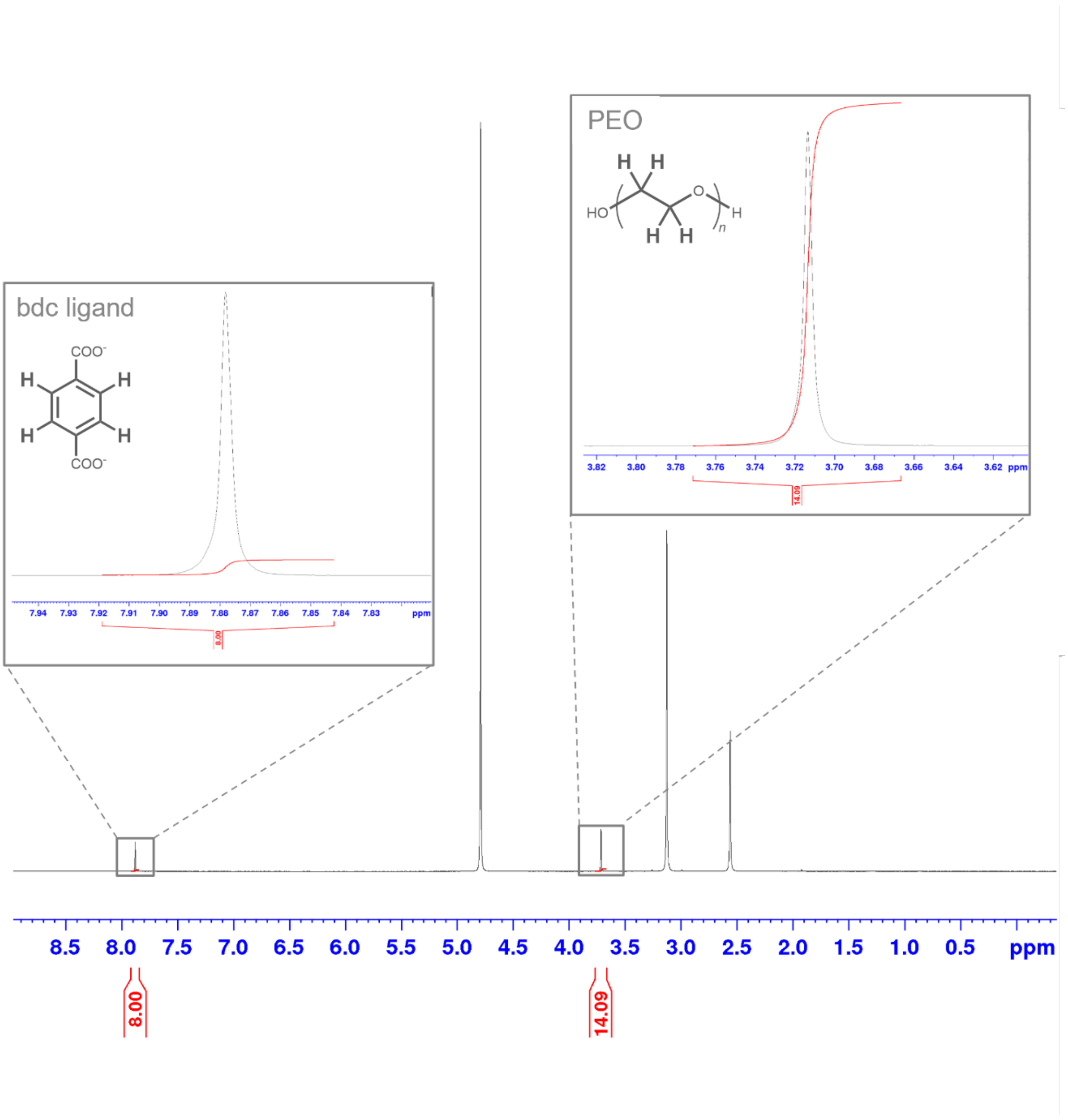


**Supplementary Fig. 22.**

$^{1}H$ NMR spectrum of **1/PEO4M$_{1/1}$** after washing (500 MHz, $D_2O$/EDTA-4Na). Based on the integral ratio between the signals corresponding to **PEO4M** (3.71 ppm) and bdc ligand (7.88 ppm), the amount of remaining **PEO4M** was calculated to be 0.25 g/g.

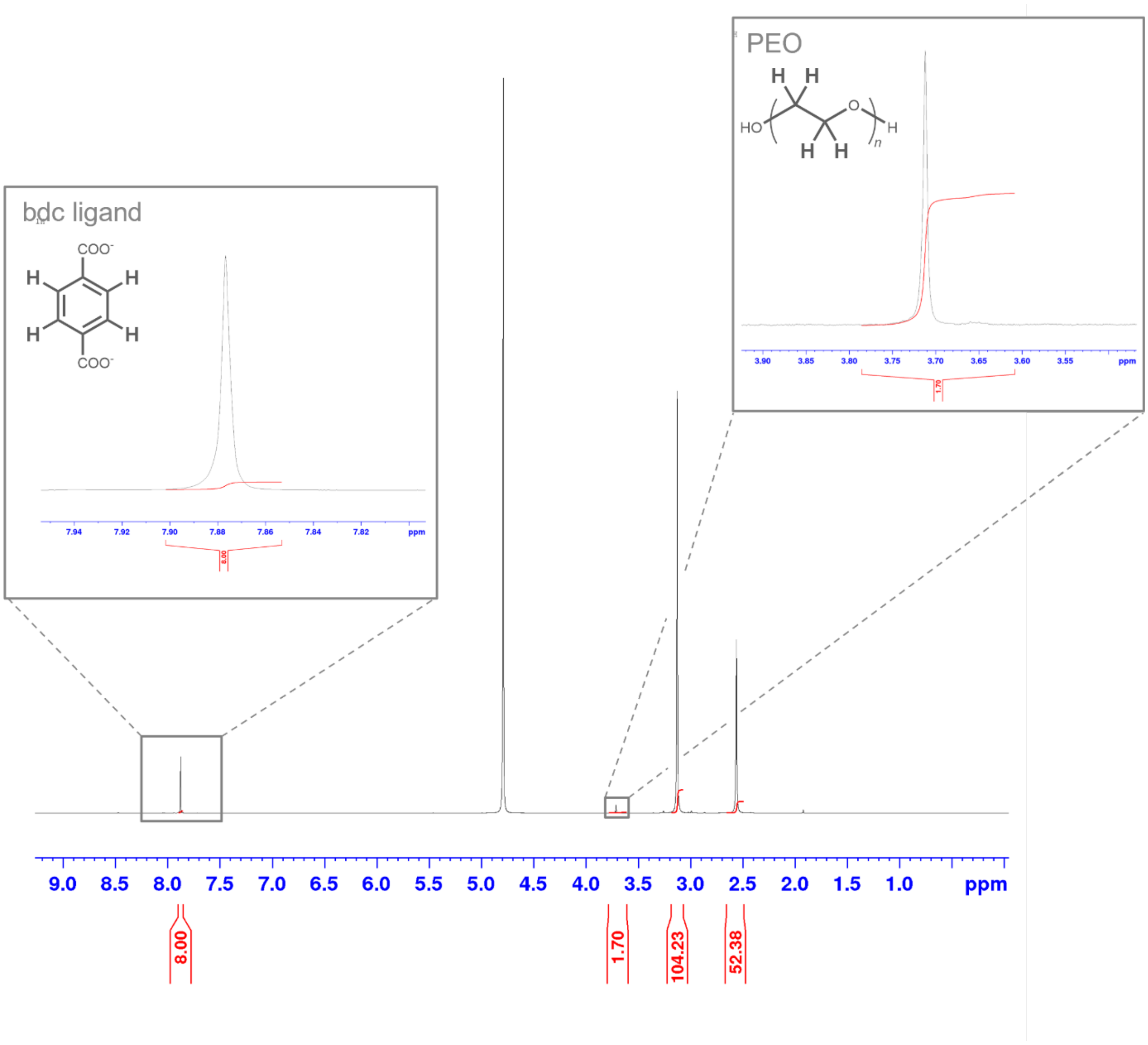


**Supplementary Fig. 23.**

$^{1}$H NMR spectrum of **1**/**PEO2k$_{1/1}$** after washing (500 MHz, $D_2O$/EDTA-4Na). Based on the integral ratio between the signals corresponding to **PEO2k** (3.71 ppm) and bdc ligand (7.88 ppm), the amount of remaining **PEO2k** was calculated to be 0.031 g/g.

**3. Uniaxial tensile stress-strain analysis on the 1/PEO composites**

We prepared thick films of **PEO200k**, **1/PEO200k$_{1/20}$** and **1/PEO200k$_{1/10}$** (Supplementary Fig. 24a) and measured their tensile properties to investigate the effect of the polythreading structure on bulk mechanical property. Uniaxial tensile tests were performed using dog-bone specimens (2 mm width, ~100 μm thickness) punched out from the films. **1/PEO200k$_{1/10}$** film showed 1.5-fold increase of elastic modulus compared to the **PEO200k** film (Supplementary Figs. 24b,c) at 25 °C. In addition, slight increase in the stress at yield and decrease in the maximum elongation length were observed (Supplementary Table 2), indicating that the addition of **1** crystals in PEO gives an effect on the material mechanical properties. Although this observation does not conflict to our hypothesis that physical cross-linking is formed through the polypseudoMOFaxane architecture, we cannot rule out the possibility of so-called filler effect that **1** particles contribute the hardening of PEO matrix as a filler. Furthermore, PEO adopts a crystalline state below its melting temperature (63 °C), and therefore the tensile properties measured at 25 °C (Supplementary Fig. 24) may primarily reflect the mechanical features of PEO crystalline domains rather than the contribution of topological crosslinking. In this context, we conducted the tensile tests at 70 °C and 77 °C, which are higher than melting temperature of PEO (Supplementary Fig. 25 and Supplementary Table 3). The results clearly showed the differences between the pristine **PEO200k** and **1/PEO200k$_{1/10}$** composite films and provided a characteristic feature that can be explained by the polypseudoMOFaxane architecture.

Despite the measurement temperatures being above the melting temperature of PEO, the pristine PEO200k film exhibited an elastic modulus that can be attributed to the entanglement effect of long PEO chains. At 70°C, the **1/PEO200k$_{1/10}$** composite film displayed a 1.2-fold larger elastic modulus than that of the pristine **PEO200k** film (Supplementary Fig. 25a), which can be attributed to the contribution of topological crosslinking by MOFs. Interestingly, at 77 °C, this trend was reversed, with the elastic modulus of the **1/PEO200k$_{1/10}$** film becoming smaller than that of the pristine **PEO200k** film (Supplementary Fig. 25b). This finding rules out the possibility of the filler effect and suggests the presence of sliding motion that is promoted at such high temperature. From the slow *cp*-to-*op* deformation rate in Fig. 3b, it is considered the sliding motion of **1** microcrystals on PEO is extremely slow and unlikely to be manifested in such mechanical tests at low temperature (~25 °C). In our previous work, we reported that the effective diffusion constant of **PEO20k** in a MOF that has similar narrow channel with the diameter of 0.57 nm is $3.1 \times 10^{-14}$ m$^2$/s (40 °C)[5]. This is $10^4$ times slower than the diffusion constant of CD of the conventional polyrotaxane in solution, which has been experimentally determined to be $1.1 \times 10^{-10}$ m$^2$/s using quasi-elastic neutron scattering (QENS) measurements (30 °C)[6]. On the other hand, at higher temperature (~77 °C), the sliding motion could be promoted, causing the elastic modulus of the composite film to decrease.

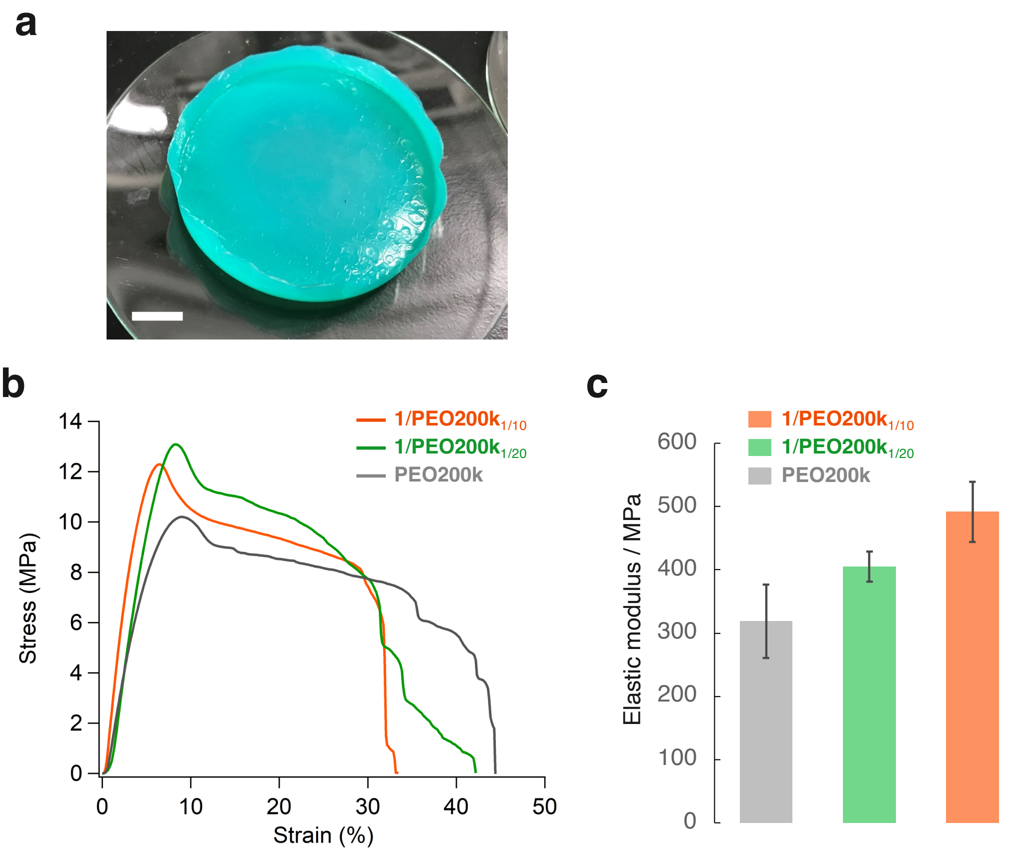


**Supplementary Fig. 24.**

**a,** A photograph of the **1/PEO200k$_{1/10}$** composite film. Scale bar: 10 mm. **b,** Typical stress-strain curves of the film **PEO200k**, **1/PEO200k$_{1/20}$** and **1/PEO200k$_{1/10}$** at 25 °C. **c,** The elastic modulus of **PEO200k** (gray), **1/PEO200k$_{1/20}$** (green) and **1/PEO200k$_{1/10}$** (orange) in the tensile analysis (Supplementary Table 2). Data are presented as mean ± SD.

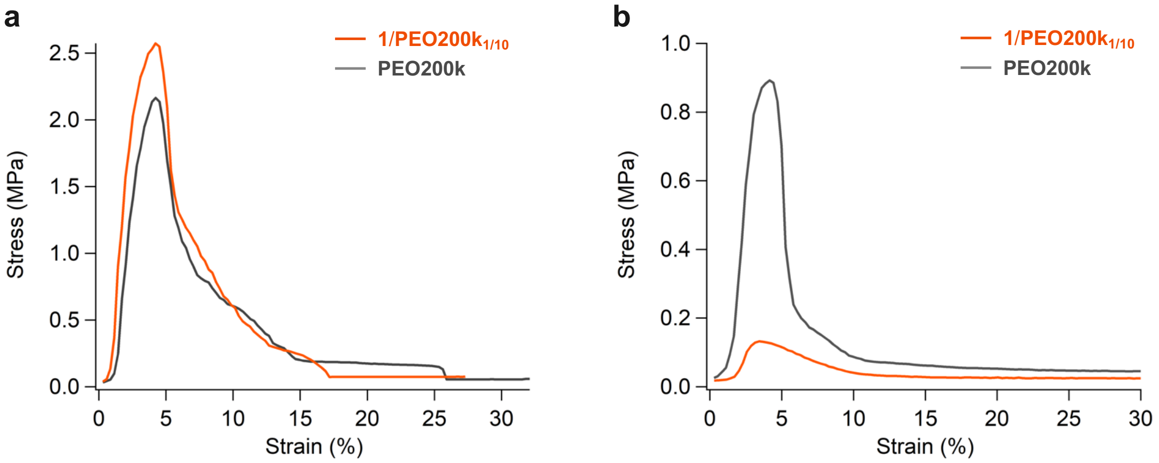


**Supplementary Fig. 25.**

Typical stress-strain curves of the **PEO200k** (black) and **1/PEO200k$_{1/10}$** (orange) films at **a,** 70 °C, and **b,** 77 °C.

**Supplementary Table 2.**
Tensile properties of **PEO200k**, **1/PEO200k$_{1/20}$**, and **1/PEO200k$_{1/10}$** film at 25 °C. Data are presented as mean ± SD.

| Sample | Elastic modulus (MPa) | Stress at yield (MPa) | Stress at break (MPa) | Elongation at break |
|---|---|---|---|---|
| **PEO200k** ($N = 8$) | 319 ± 58 | 10.6 ± 1.9 | 6.6 ± 1.6 | 44.1 ± 6.7% |
| **1/PEO200k$_{1/20}$** ($N = 3$) | 405 ± 24 | 13.1 ± 0.7 | 8.4 ± 1.0 | 42.2 ± 3.3% |
| **1/PEO200k$_{1/10}$** ($N = 6$) | 492 ± 47 | 12.7 ± 0.9 | 7.6 ± 0.7 | 39.0 ± 8.1% |

**Supplementary Table 3.**
Temperature dependence of elastic modulus of **PEO200k** and **1/PEO200k$_{1/10}$** film. Data are presented as mean ± SD ($N = 3$).

| Temperature | **PEO200k** | **1/PEO200k$_{1/10}$** |
|---|---|---|
| 25 °C | 319 ± 58 MPa | 492 ± 47 MPa |
| 70 °C | 113 ± 15 MPa | 139 ± 33 MPa |
| 77 °C | 48.4 ± 17.0 MPa | 13.3 ± 13.2 MPa |

## V. Supplementary References


1. Sakata, Y. et al. Shape-Memory Nanopores Induced in Coordination Frameworks by Crystal Downsizing. *Science* **339**, 193–196 (2013).
2. Le Ouay, B. et al. Selective sorting of polymers with different terminal groups using metal-organic frameworks. *Nat. Commun.* **9**, 3635 (2018).
3. Friedman, H. L. Kinetics of thermal degradation of char-forming plastics from thermogravimetry. Application to phenolic plastic. *J. Polym. Sci. C* **6**, 193 (1964).
4. Vyazovskin, S., Sbirrazzuoli, N. Isoconversional Kinetic Analysis of Thermally Simulated Processes in Polymers. *Macromol. Rapid Commun.* **27**, 1515 (2006).
5. Oe, N., Hosono, N. & Uemura, T. Revisiting molecular adsorption: unconventional uptake of polymer chains from solution into sub-nanoporous media. *Chem. Sci.* **12**, 12576–12586 (2021).
6. Yasuda, Y. et al. Molecular Dynamics of Polyrotaxane in Solution Investigated by Quasi-Elastic Neutron Scattering and Molecular Dynamics Simulation: Sliding Motion of Rings on Polymer. *J. Am. Chem. Soc.* **141**, 9655–9663 (2019).
